# Poly[[1-(2-pyrid­yl)ethanone-κ^2^ *N*,*O*]di-μ_2_-thio­cyanato-κ^2^ *N*:*S*;κ^2^ *S*:*N*-cadmium(II)]

**DOI:** 10.1107/S1600536810025225

**Published:** 2010-07-03

**Authors:** Jian-Ying Miao

**Affiliations:** a Department of Chemistry and Chemical Engineering, Baoji University of Arts and Sciences, Baoji 721007, People’s Republic of China

## Abstract

In the title compound, [Cd(NCS)_2_(C_7_H_7_NO)]_*n*_, the Cd^2+^ ion is six-coordinated by one *N*,*O*-bidentate 1-(2-pyridyletahanone ligand, two *N*-bonded thio­cyanate ions and two *S*-bonded thio­cyanate ions. In the resulting distorted CdOS_2_N_3_ octa­hedron, the N atoms adopt a *fac* arrangement. The bridging thio­cyanate ions lead to infinite sheets oriented parallel to (101) in the crystal structure.

## Related literature

For background to cadmium complexes, see: Banerjee *et al.* (2005 ▶); Shi *et al.* (2004 ▶); Ercan *et al.* (2004 ▶); Reger *et al.* (2002 ▶); Ghosh *et al.* (2007 ▶). For related cadmium complexes with thio­cyanate bridges, see: Zhao *et al.* (2006 ▶); Bigoli *et al.* (1972 ▶); Taniguchi *et al.* (1986 ▶); Marsh *et al.* (1995 ▶); Yang *et al.* (2001 ▶).
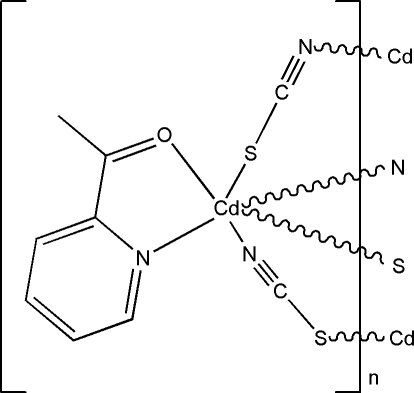


## Experimental

### 

#### Crystal data

[Cd(NCS)_2_(C_7_H_7_NO)]
*M* *_r_* = 349.70
Monoclinic, 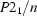

*a* = 12.3511 (12) Å
*b* = 7.6540 (8) Å
*c* = 12.5636 (12) Å
β = 97.045 (1)°
*V* = 1178.7 (2) Å^3^
*Z* = 4
Mo *K*α radiation
μ = 2.19 mm^−1^
*T* = 298 K
0.27 × 0.27 × 0.22 mm

#### Data collection

Bruker SMART CCD diffractometer
Absorption correction: multi-scan (*SADABS*; Sheldrick, 1996 ▶) *T*_min_ = 0.580, *T*_max_ = 0.645
7522 measured reflections
2559 independent reflections
2234 reflections with *I* > 2σ(*I*)
*R*_int_ = 0.021

#### Refinement

*R*[*F* ^2^ > 2σ(*F* ^2^)] = 0.022
*wR*(*F* ^2^) = 0.054
*S* = 1.06
2559 reflections
146 parameters
H-atom parameters constrained
Δρ_max_ = 0.29 e Å^−3^
Δρ_min_ = −0.64 e Å^−3^

### 

Data collection: *SMART* (Bruker, 1998 ▶); cell refinement: *SAINT* (Bruker, 1998 ▶); data reduction: *SAINT*; program(s) used to solve structure: *SHELXS97* (Sheldrick, 2008 ▶); program(s) used to refine structure: *SHELXL97* (Sheldrick, 2008 ▶); molecular graphics: *SHELXTL* (Sheldrick, 2008 ▶); software used to prepare material for publication: *SHELXTL*.

## Supplementary Material

Crystal structure: contains datablocks global, I. DOI: 10.1107/S1600536810025225/hb5520sup1.cif

Structure factors: contains datablocks I. DOI: 10.1107/S1600536810025225/hb5520Isup2.hkl

Additional supplementary materials:  crystallographic information; 3D view; checkCIF report

## Figures and Tables

**Table 1:** Selected bond lengths (Å)

Cd1—N2^i^	2.271 (2)
Cd1—N3^ii^	2.314 (2)
Cd1—N1	2.336 (2)
Cd1—O1	2.4571 (19)
Cd1—S2	2.6235 (8)
Cd1—S1	2.7269 (7)

Symmetry codes: (i) 
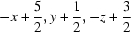
; (ii) 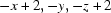.

**Fig. 1.**
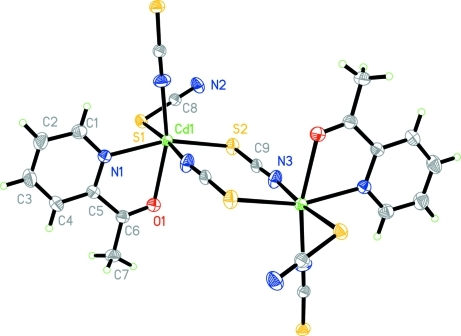
A fragment of (I), showing 30% displacement ellipsoids (arbitrary spheres for the H atoms).

**Fig. 2.**
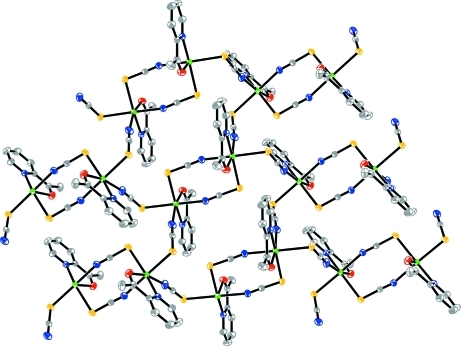
The two-dimensional sheet of (I).

## supplementary crystallographic information

### Comment

Considerable attention has been focused on the cadmium(II) complexes with multidentate ligands (Banerjee *et al.*, 2005; Shi *et al.*, 2004; Ercan *et al.*, 2004; Reger *et al.*, 2002; Ghosh *et al.*, 2007). As an extension of the work on the structural characterization of such complexes, the title new polynuclear cadmium(II) complex is reported here.

The title compound is a thiocyanate-bridged polynuclear cadmium(II) complex, as shown in Fig. 1. Each Cd atom is six-coordinated by one O and one N atoms of 2-acetylpyridine (*L*), and by two N and two S atoms from four thiocyanate ligands, forming an octahedral geometry. The bond lengths in the octahedral coordination are comparable with those reported in similar cadmium structures with thiocyanate bridges (Zhao *et al.*, 2006; Bigoli *et al.*, 1972; Taniguchi *et al.*, 1986; Marsh *et al.*, 1995; Yang *et al.*, 2001). The adjacent two CdL units are linked by two thiocyanate ligands, forming a dimer. The dimers are further linked by thiocyanate ligands, forming a two-dimensional sheet, as shown in Fig. 2.

### Experimental

2-Acetylpyridine (1 mmol, 121 mg), ammonium thiocyanate (2 mmol, 152 mg), and Cd(NO_3_)_2_.4H_2_O (1 mmol, 308 mg) were dissolved in MeOH (80 ml). The mixture was stirred at room temperature for 1 h to give a colorless solution. The resulting solution was kept in air for a week, and colorless blocks of (I) were formed as the solvent slowly evaporated.

### Refinement

H atoms were placed in idealized positions and constrained to ride on their parent atoms, with C—H distances in the range 0.93–0.96 Å, and with *U*_iso_(H) = 1.2 or 1.5*U*_eq_(C).

### Crystal data

**Table:**

[Cd(NCS)_2_(C_7_H_7_NO)]	*F*(000) = 680
*M**_r_* = 349.70	*D*_x_ = 1.971 Mg m^−^^3^
Monoclinic, *P*2_1_/*n*	Mo *K*α radiation, λ = 0.71073 Å
Hall symbol: -P 2yn	Cell parameters from 3922 reflections
*a* = 12.3511 (12) Å	θ = 2.4–28.3°
*b* = 7.6540 (8) Å	µ = 2.19 mm^−^^1^
*c* = 12.5636 (12) Å	*T* = 298 K
β = 97.045 (1)°	Block, colorless
*V* = 1178.7 (2) Å^3^	0.27 × 0.27 × 0.22 mm
*Z* = 4	

### Data collection

**Table:**

Bruker SMART CCD diffractometer	2559 independent reflections
Radiation source: fine-focus sealed tube	2234 reflections with *I* > 2σ(*I*)
graphite	*R*_int_ = 0.021
ω scans	θ_max_ = 27.0°, θ_min_ = 2.5°
Absorption correction: multi-scan (*SADABS*; Sheldrick, 1996)	*h* = −14→15
*T*_min_ = 0.580, *T*_max_ = 0.645	*k* = −7→9
7522 measured reflections	*l* = −16→16

### Refinement

**Table:**

Refinement on *F*^2^	Primary atom site location: structure-invariant direct methods
Least-squares matrix: full	Secondary atom site location: difference Fourier map
*R*[*F*^2^ > 2σ(*F*^2^)] = 0.022	Hydrogen site location: inferred from neighbouring sites
*wR*(*F*^2^) = 0.054	H-atom parameters constrained
*S* = 1.06	*w* = 1/[σ^2^(*F*_o_^2^) + (0.0236*P*)^2^ + 0.5142*P*] where *P* = (*F*_o_^2^ + 2*F*_c_^2^)/3
2559 reflections	(Δ/σ)_max_ < 0.001
146 parameters	Δρ_max_ = 0.29 e Å^−^^3^
0 restraints	Δρ_min_ = −0.64 e Å^−^^3^

### Special details

**Table:**

Geometry. All e.s.d.'s (except the e.s.d. in the dihedral angle between two l.s. planes) are estimated using the full covariance matrix. The cell e.s.d.'s are taken into account individually in the estimation of e.s.d.'s in distances, angles and torsion angles; correlations between e.s.d.'s in cell parameters are only used when they are defined by crystal symmetry. An approximate (isotropic) treatment of cell e.s.d.'s is used for estimating e.s.d.'s involving l.s. planes.
Refinement. Refinement of *F*^2^ against ALL reflections. The weighted *R*-factor *wR* and goodness of fit *S* are based on *F*^2^, conventional *R*-factors *R* are based on *F*, with *F* set to zero for negative *F*^2^. The threshold expression of *F*^2^ > σ(*F*^2^) is used only for calculating *R*-factors(gt) *etc*. and is not relevant to the choice of reflections for refinement. *R*-factors based on *F*^2^ are statistically about twice as large as those based on *F*, and *R*- factors based on ALL data will be even larger.

### Fractional atomic coordinates and isotropic or equivalent isotropic displacement parameters (Å^2^)

**Table:**

	*x*	*y*	*z*	*U*_iso_*/*U*_eq_	
Cd1	1.056450 (14)	0.09491 (3)	0.785436 (13)	0.03489 (7)	
N1	0.95994 (16)	0.3107 (3)	0.68209 (16)	0.0364 (5)	
N2	1.2939 (2)	−0.2353 (4)	0.67082 (19)	0.0543 (7)	
N3	1.0159 (2)	−0.1892 (3)	1.06320 (18)	0.0517 (6)	
O1	0.87057 (15)	−0.0002 (3)	0.71826 (16)	0.0502 (5)	
S1	1.12429 (5)	−0.00856 (10)	0.59676 (5)	0.04115 (16)	
S2	1.10322 (6)	−0.21406 (10)	0.86912 (5)	0.04669 (18)	
C1	1.0029 (2)	0.4653 (4)	0.6646 (2)	0.0477 (7)	
H1	1.0753	0.4858	0.6914	0.057*	
C2	0.9446 (3)	0.5976 (4)	0.6082 (3)	0.0598 (8)	
H2	0.9771	0.7048	0.5975	0.072*	
C3	0.8386 (3)	0.5672 (4)	0.5688 (3)	0.0596 (9)	
H3	0.7976	0.6539	0.5308	0.072*	
C4	0.7924 (2)	0.4074 (4)	0.5857 (2)	0.0488 (7)	
H4	0.7201	0.3851	0.5592	0.059*	
C5	0.8547 (2)	0.2807 (3)	0.64241 (18)	0.0353 (5)	
C6	0.8117 (2)	0.1048 (4)	0.6665 (2)	0.0401 (6)	
C7	0.6955 (2)	0.0612 (4)	0.6271 (2)	0.0562 (8)	
H7A	0.6826	0.0793	0.5510	0.084*	
H7B	0.6479	0.1352	0.6620	0.084*	
H7C	0.6816	−0.0588	0.6430	0.084*	
C8	1.2247 (2)	−0.1410 (4)	0.64196 (19)	0.0365 (6)	
C9	1.0505 (2)	−0.1971 (3)	0.9824 (2)	0.0366 (5)	

### Atomic displacement parameters (Å^2^)

**Table:**

	*U*^11^	*U*^22^	*U*^33^	*U*^12^	*U*^13^	*U*^23^
Cd1	0.03050 (11)	0.04039 (12)	0.03362 (10)	−0.00046 (8)	0.00330 (7)	0.00215 (8)
N1	0.0353 (11)	0.0383 (12)	0.0356 (10)	0.0008 (10)	0.0050 (9)	−0.0021 (9)
N2	0.0480 (14)	0.0677 (18)	0.0469 (13)	0.0216 (13)	0.0040 (11)	0.0042 (12)
N3	0.0677 (16)	0.0464 (14)	0.0445 (13)	0.0133 (13)	0.0216 (12)	0.0088 (11)
O1	0.0408 (11)	0.0483 (12)	0.0601 (12)	−0.0025 (10)	0.0012 (9)	0.0110 (10)
S1	0.0385 (3)	0.0497 (4)	0.0352 (3)	0.0111 (3)	0.0045 (3)	0.0026 (3)
S2	0.0606 (4)	0.0451 (4)	0.0365 (3)	0.0130 (4)	0.0139 (3)	0.0011 (3)
C1	0.0477 (16)	0.0435 (16)	0.0505 (16)	−0.0066 (14)	0.0005 (13)	−0.0039 (13)
C2	0.072 (2)	0.0399 (17)	0.065 (2)	−0.0077 (16)	−0.0006 (17)	0.0078 (15)
C3	0.066 (2)	0.0461 (19)	0.064 (2)	0.0109 (16)	−0.0067 (16)	0.0081 (15)
C4	0.0454 (16)	0.0511 (18)	0.0474 (15)	0.0063 (14)	−0.0039 (13)	0.0008 (13)
C5	0.0352 (13)	0.0414 (14)	0.0295 (11)	0.0025 (11)	0.0042 (10)	−0.0051 (10)
C6	0.0382 (14)	0.0470 (16)	0.0355 (13)	−0.0021 (12)	0.0055 (11)	−0.0011 (12)
C7	0.0403 (16)	0.073 (2)	0.0530 (17)	−0.0140 (15)	−0.0014 (13)	0.0057 (16)
C8	0.0355 (13)	0.0442 (15)	0.0303 (12)	0.0012 (12)	0.0064 (10)	−0.0017 (11)
C9	0.0394 (14)	0.0323 (14)	0.0374 (13)	0.0059 (11)	0.0025 (11)	0.0057 (11)

### Geometric parameters (Å, °)

**Table:**

Cd1—N2^i^	2.271 (2)	S2—C9	1.642 (3)
Cd1—N3^ii^	2.314 (2)	C1—C2	1.386 (4)
Cd1—N1	2.336 (2)	C1—H1	0.9300
Cd1—O1	2.4571 (19)	C2—C3	1.362 (5)
Cd1—S2	2.6235 (8)	C2—H2	0.9300
Cd1—S1	2.7269 (7)	C3—C4	1.377 (4)
N1—C1	1.326 (4)	C3—H3	0.9300
N1—C5	1.353 (3)	C4—C5	1.380 (4)
N2—C8	1.143 (3)	C4—H4	0.9300
N2—Cd1^iii^	2.271 (2)	C5—C6	1.492 (4)
N3—C9	1.149 (3)	C6—C7	1.497 (4)
N3—Cd1^ii^	2.314 (2)	C7—H7A	0.9600
O1—C6	1.217 (3)	C7—H7B	0.9600
S1—C8	1.648 (3)	C7—H7C	0.9600
			
N2^i^—Cd1—N3^ii^	90.44 (9)	C2—C1—H1	118.5
N2^i^—Cd1—N1	94.28 (9)	C3—C2—C1	118.5 (3)
N3^ii^—Cd1—N1	90.80 (8)	C3—C2—H2	120.8
N2^i^—Cd1—O1	162.00 (9)	C1—C2—H2	120.8
N3^ii^—Cd1—O1	86.32 (8)	C2—C3—C4	119.6 (3)
N1—Cd1—O1	68.10 (7)	C2—C3—H3	120.2
N2^i^—Cd1—S2	106.62 (7)	C4—C3—H3	120.2
N3^ii^—Cd1—S2	92.30 (6)	C3—C4—C5	119.3 (3)
N1—Cd1—S2	158.83 (6)	C3—C4—H4	120.4
O1—Cd1—S2	91.21 (5)	C5—C4—H4	120.4
N2^i^—Cd1—S1	92.80 (6)	N1—C5—C4	121.3 (3)
N3^ii^—Cd1—S1	174.86 (6)	N1—C5—C6	115.4 (2)
N1—Cd1—S1	84.98 (5)	C4—C5—C6	123.3 (2)
O1—Cd1—S1	89.38 (5)	O1—C6—C5	120.0 (2)
S2—Cd1—S1	90.60 (2)	O1—C6—C7	121.1 (3)
C1—N1—C5	118.4 (2)	C5—C6—C7	119.0 (2)
C1—N1—Cd1	122.40 (18)	C6—C7—H7A	109.5
C5—N1—Cd1	119.10 (17)	C6—C7—H7B	109.5
C8—N2—Cd1^iii^	173.0 (2)	H7A—C7—H7B	109.5
C9—N3—Cd1^ii^	164.7 (2)	C6—C7—H7C	109.5
C6—O1—Cd1	117.39 (18)	H7A—C7—H7C	109.5
C8—S1—Cd1	100.23 (9)	H7B—C7—H7C	109.5
C9—S2—Cd1	100.65 (9)	N2—C8—S1	178.2 (3)
N1—C1—C2	122.9 (3)	N3—C9—S2	177.8 (2)
N1—C1—H1	118.5		

Symmetry codes: (i) −*x*+5/2, *y*+1/2, −*z*+3/2; (ii) −*x*+2, −*y*, −*z*+2; (iii) −*x*+5/2, *y*−1/2, −*z*+3/2.
